# Adapting the Dejour classification of trochlear dysplasia from qualitative radiograph‐ and CT‐based assessments to quantitative MRI‐based measurements

**DOI:** 10.1002/ksa.12539

**Published:** 2024-11-18

**Authors:** David H. Dejour, Edoardo Giovanetti de Sanctis, Jacobus H. Müller, Etienne Deroche, Tomas Pineda, Amedeo Guarino, Cécile Toanen, Patellofemoral Imaging Group, Andrea Amarossi, Alexandre Baujard, Nicolas Cance, Julien Dartus, Paolo Ferrua, Stefano Pasqualotto, Francesco Puglia, Sophie Putman, Mo Saffarini

**Affiliations:** ^1^ Lyon‐Ortho‐Clinic, Clinique de la Sauvegarde, Ramsay Santé Lyon France; ^2^ ReSurg SA Nyon Switzerland; ^3^ Centre Orthopédique de Dracy‐Le‐Fort Dracy‐Le‐Fort France; ^4^ Département de Chirurgie Orthopédique Polyclinique Bordeaux Nord Aquitaine Bordeaux France; ^5^ Department of Orthopaedics IRCCS Ospedale Sacro Cuore Don Calabria, Negrar di Valpolicella Verona Italy; ^6^ Département Universitaire de Chirurgie Orthopédique et Traumatologique CHU Lille University of Lille Lille France; ^7^ Dipartimento Scienze Biomediche per la Salute Università degli Studi di Milano Milano Italy; ^8^ UOC Prima Clinica Ortopedica, ASST Gaetano Pini‐CTO Milano Milano Italy

**Keywords:** bump, lateral trochlear inclination, magnetic resonance imaging, objective patellar instability, sulcus angle, trochlear dysplasia

## Abstract

**Purpose:**

To adapt the current D. Dejour trochlear dysplasia classification (v2.0) to only rely on quantitative magnetic resonance imaging (MRI) measurements (v3.0) to maximize objectivity and repeatability.

**Methods:**

A consecutive series of adult knee MRIs were divided into objective patellar instability (OPI, *n* = 127) or controls (*n* = 103; isolated meniscal tears) and postprocessed with multiplanar reconstruction (MPR) to standardize the sagittal plane and ensure true lateral views. Thresholds for sulcus angle, lateral trochlear inclination (LTI) and central bump were established using regression tree models to distinguish OPI from controls. The sensitivity and specificity of sulcus angle and LTI combinations to diagnose OPI were then evaluated, and the combination yielding the highest sensitivity was selected as basis for trochlear dysplasia classification. Finally, sulcus angle and LTI measurability and presence of a central bump >5 mm were used to grade dysplasia as low, moderate or high.

**Results:**

The regression tree models produced thresholds of ≥157° for sulcus angle and <14° for LTI to distinguish OPI from controls. ‘Sulcus angle ≥157° OR LTI < 14°’ yielded the highest sensitivity (87%) to diagnose OPI. The quantitative MRI classification was sulcus angle <157° **AND** LTI ≥ 14° for Type 0 (No dysplasia); (sulcus angle ≥ 157° **OR** LTI < 14°) **AND** central bump <5 mm for Type 1 (Low‐grade dysplasia); (sulcus angle **OR** LTI are ‘unmeasurable’) **AND** central bump <5 mm for Type 2 (Moderate‐grade dysplasia); (sulcus angle ≥ 157° **OR** ‘unmeasurable’ **OR** LTI < 14° **OR** ‘unmeasurable’) **AND** central bump ≥5 mm for Type 3 (High‐grade dysplasia).

**Conclusion:**

This MRI classification depends exclusively on quantitative measurements, has excellent interobserver agreement, and yields high sensitivity to diagnose OPI. The MRI imaging protocol with MPR mode and standardized measurements could be quickly adopted and correctly applied by clinicians worldwide in any type of institution to determine the ideal treatment plan.

**Level of Evidence:**

Level III.

**Abbreviations**
3D: three‐dimensional
AC: agreement coefficient
AFCL: anterior femoral cortex line
C.I.: confidence interval
CT: computed tomography
CTO: cranial trochlear orientation
FP: false positive
ICC: intraclass correlation coefficient
IQR: interquartile range
L: lateral
LTC: lateral trochlear cartilage
LTI: lateral trochlear inclination
M: medial
mm: millimetre
MPR: multiplanar reconstruction
MRI: magnetic resonance imaging
MTC: medial trochlear cartilage
OPI: objective patellar instability
PBCL: posterior bicondylar line
SD: standard deviation
TGC: trochlear groove cartilage
TN: true negative

## INTRODUCTION

Diagnosing and classifying trochlear dysplasia remains challenging despite numerous attempts to describe and develop reliable methods [27]. Brattström (1964) [1] first described trochlear dysplasia using sulcus angle measurements on skyline radiographs with the knee in 30° flexion. Hepp (1982) later classified trochlear dysplasia based on a qualitative assessment of the morphology on ‘tangential’ radiographs [16]. The first classification by Henri Dejour (v1.0, 1987) [5, 6] relied primarily on the presence and level of the crossing sign on true lateral knee radiographs, which was further developed by David Dejour (v2.0, 1998) [3, 31] to inspect for a crossing sign, a supratrochlear spur and a double contour sign by cross‐referencing true lateral radiographs with axial computed tomography (CT).

A recent systematic review confirmed that the D. Dejour trochlear dysplasia classification is still the most commonly used [26]. However, clinicians often don't follow the correct imaging protocols because it is challenging to align the posterior condyles to get true lateral radiographs. Additionally, nine of the 11 eligible studies used only one imaging modality. As magnetic resonance imaging (MRI) became more widely used to diagnose patellofemoral disorders, further attempts were made to develop alternative qualitative classifications that can be subjective and lead to poor repeatability and agreement [8, 18, 29]. The advantages of using only MRI include postprocessing methods to ensure well‐oriented images irrespective of patient position during imaging, assessment of all related parameters in three‐dimensional (3D) space, and visualization of cartilage to enable examination of cartilage‐based landmarks to assess joint conformity.

The purpose of the present study was to adapt the current D. Dejour trochlear dysplasia classification (v2.0) to only use MRI to facilitate the process and only rely on quantitative measurements to maximize objectivity and repeatability. The clinical relevance is that an MRI classification of trochlear dysplasia (v3.0) based purely on quantitative parameters would be easily adopted and correctly applied by clinicians worldwide and in all types of institutions.

## MATERIALS AND METHODS

### Cohort

**Figure 1:**
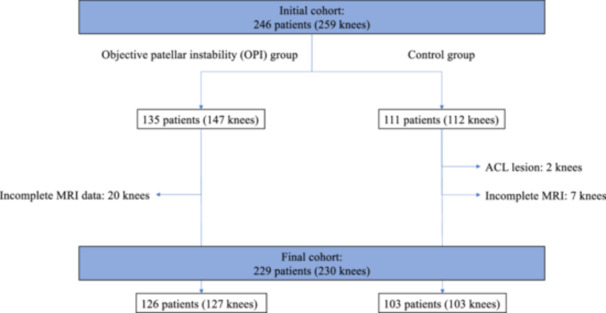
Flowchart of the study cohort of knees in the objective patellar instability (OPI) and control groups.

In this retrospective comparative analysis, the authors reviewed records of all adult men and women who received an MRI of the knee between 2019 and 2022 at the senior authors' (DHD) centre. The objective patellar instability (OPI) group were patients with a history of more than two episodes of documented lateral patellar dislocation and no previous knee surgery, and the control group patients with isolated meniscal tears and no history of patellofemoral disorders (anterior knee pain, patellar dislocation) or knee surgery. Patients were excluded if there were signs of osteoarthritis in any of the three compartments or signs of ligament injuries. In the initial cohort of 246 patients (OPI, 135; Control, 111) eligible for inclusion, 27 had incomplete MRI data and two had anterior cruciate ligament lesions, leaving a final cohort of 229 patients (OPI, 126; Control, 103) (Figure 1, Table 1). Each patient gave written informed consent to use their data and images for research and publishing purposes. The institutional review board approved the study in advance (IRB: COS‐RGDS‐2023‐11‐008‐DEJOUR‐D) and conducted it per the Declaration of Helsinki.

**Table 1:** Characteristics of all knees included in the analysis.

	Mean ± SD; *n* (%)	Range	*Median*	*IQR*
Group				
Control	103 (45%)			
OPI	127 (55%)			
Trochlear shape				
Concave	97 (42%)			
Flat	88 (38%)			
Convex	45 (20%)			
Sulcus angle, deg	154.1 ± 8.7	134–180	*153*	*148*–*160*
LTI, deg	14.3 ± 4.8	0–29	*14*	*11*–*18*
CTO, deg	−6.1 ± 8.2	−38–22	*−6*	*−11–−2*
Central bump				
Axial plane, mm	4.5 ± 2.6	−2–12	*4*	*3*–*6*
Sagittal plane, mm	4.6 ± 2.6	−2–12	*4*	*3*–*6*

Abbreviations: CTO, Cranial trochlear orientation; deg, degrees; IQR, interquartile range; LTI, lateral trochlear inclination; mm, millimetre; OPI, objective patella instability; SD, standard deviation.

### MRI protocol

The MRIs were obtained on a Philips Ambition 1.5‐T system, with the patient supine, and their knee slightly flexed in neutral rotation in a dedicated knee coil (Coil Knee 16ch). The following 3D DP fat‐saturated sequences (fast recovery, fast spin echo accelerated) were obtained without venous paramagnetic contrast agents:

- sagittal (repetition time, 900 ms; echo time, 102 ms; frequency phase, 252 × 250; number of excitations, one; field of view, 200 × 200 cm; Resolution, 08 × 0.8 × 0.8 mm; slices, 279; ecotrain, 24; bandwidth, 328.5 kHz)

The MRI images were then postprocessed using the multiplanar reconstruction (MPR) mode [11], allowing simultaneous displays in the axial, sagittal and coronal planes of the acquired knee images (3D sequence acquisition of an anatomical volume). The MPR mode enables standardization of knee rotation in the axial plane to ensure true lateral views—independent of patient position during the scan—by aligning the sagittal plane perpendicular to the posterior bicondylar line (PBCL). This axial plane could then be used to reconstruct the two orthogonal planes, that is, sagittal and coronal, with the knee in its manipulated orientation. A 3D sequence with a high spatial and equal resolution in all three planes provided a 3D image with cubic voxels and good‐quality views in the three planes when using the MPR mode.

### Trochlear measurement protocol

**Figure 2:**
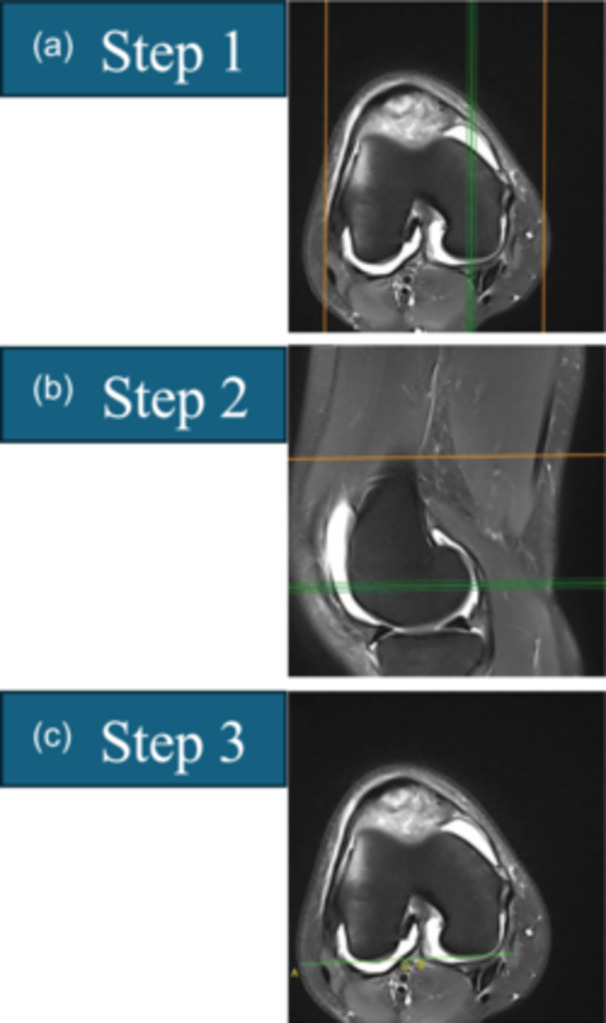
Identifying the posterior bicondylar line (PBCL). The PBCL is defined in three steps. (a) Step 1: Select the axial magnetic resonance imaging (MRI) slice that crosses the centre of the medial condyle. (b) Step 2: Select the sagittal MRI slice that crosses the most posterior point of the medial condyle. (c) Step 3: Return to the axial slice and draw the PBCL, which passes through the subchondral bone at the posterior aspect of the medial and lateral femoral condyles.

**Figure 3:**
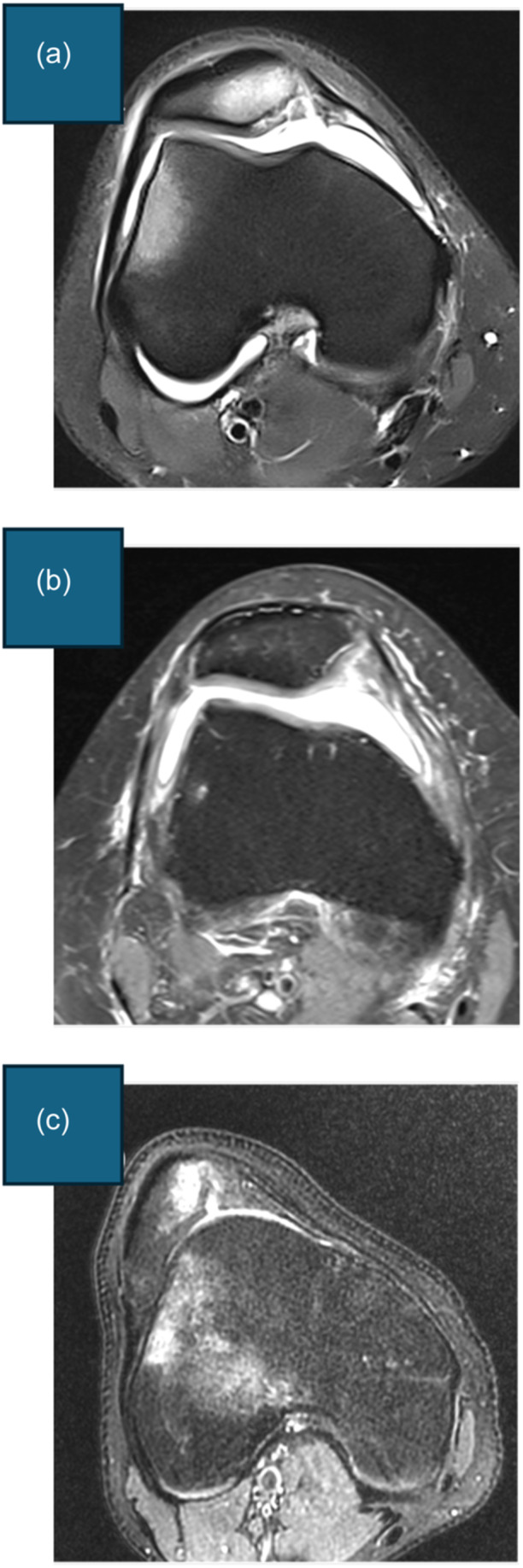
Description of trochlear shape as concave, flat or convex. Start by scrolling from cranial to caudal, select the first axial slice with the trochlear cartilage visible and showing or covering at least the entire lateral facet. The trochlear shape is described according to the cartilage contour as (a) Concave: trochlea with a sulcus, as well as medial and lateral facets. (b) Flat: trochlea with no evident sulcus nor medial facet. (c) Convex: trochlea with no sulcus nor medial facet and domed lateral facet.

**Figure 4:**
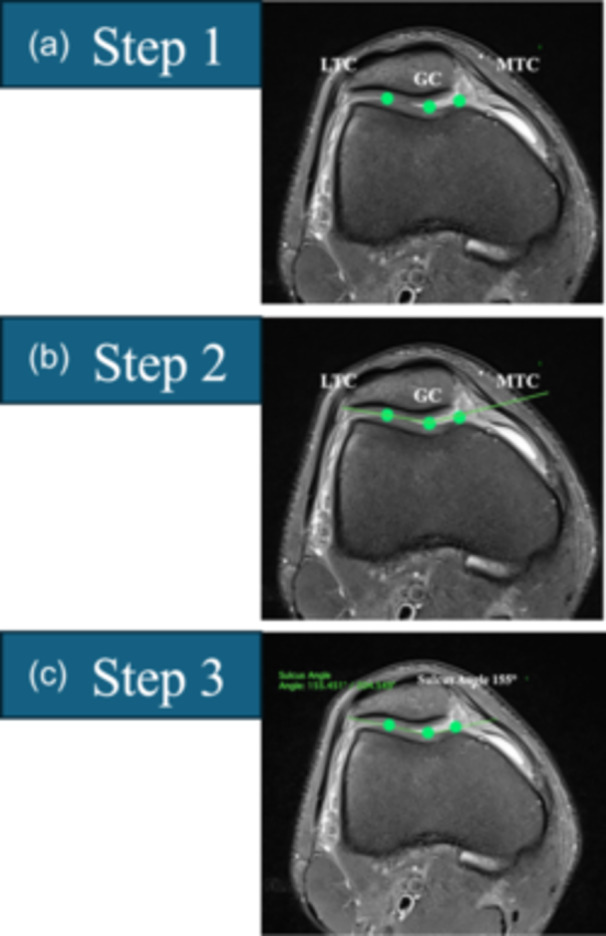
The sulcus angle is measured in three steps for concave and flat trochleae (sulcus angle is unmeasurable in convex trochlea). Start by scrolling from cranial to caudal and select the first slice where the trochlear cartilage is clearly visible showing the formation of the sulcus and the medial facet (choose the first image with the medial facet beginning to form; the medial facet need not be completely formed). (a) Step 1: Digitize the trochlear groove cartilage (TGC) point and the peaks of the lateral trochlear cartilage (LTC) and medial trochlear cartilage (MTC). (b) Step 2: Draw a straight line (LTC–TGC) between LTC and TGC and a straight line (TGC–MTC) between TGC and MTC. (c) Step 3: Measure the sulcus angle between lines LTC–TGC and TGC–MTC.

**Figure 5:**
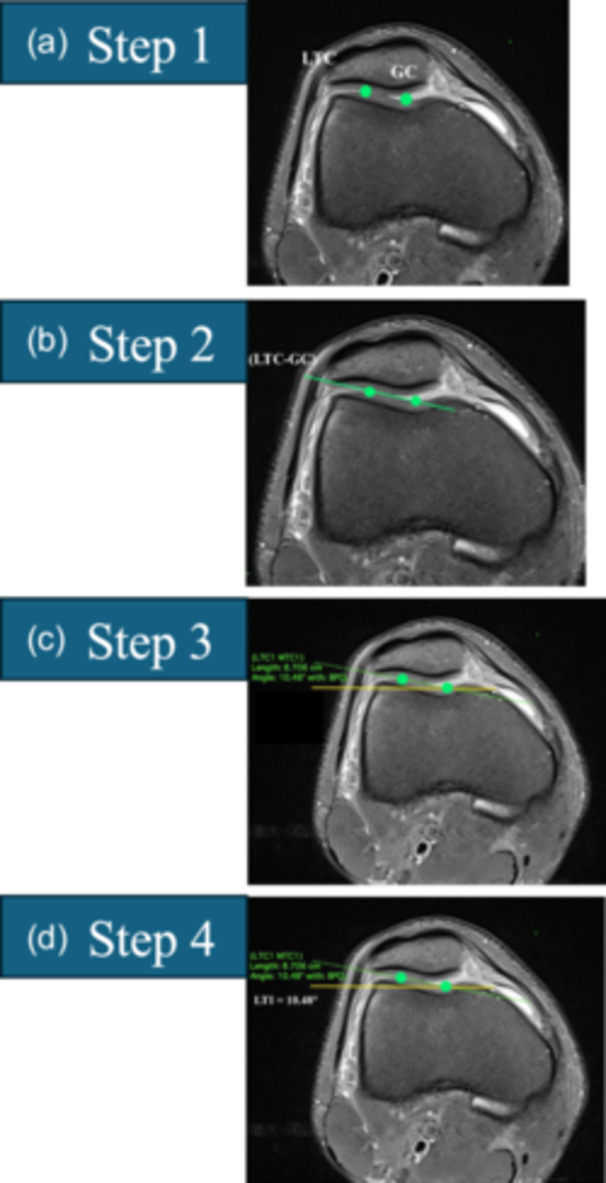
The lateral trochlear inclination (LTI) is measured in four steps for concave and flat trochleae (LTI is unmeasurable in convex trochlea). Start by scrolling from cranial to caudal and select the first slice where the trochlear cartilage is clearly visible showing the formation of the sulcus and the medial facet (choose the first image with the medial facet beginning to form; the medial facet need not be completely formed). (a) Step 1: Digitize the trochlear groove cartilage (TGC) point and the lateral trochlear cartilage (LTC) peak. (b) Step 2: Draw a straight line (LTC–TGC) between LTC and TGC. (c) Step 3: Copy and paste the posterior bicondylar line (PBCL) and translate it anteriorly to intersect point TGC. (d) Step 4: Measure the angle between lines LTC–TGC and PBCL. The angle is considered positive if LTC is anterior to the straight line. The angle is considered negative if LTC is posterior to the straight line.

**Figure 6:**
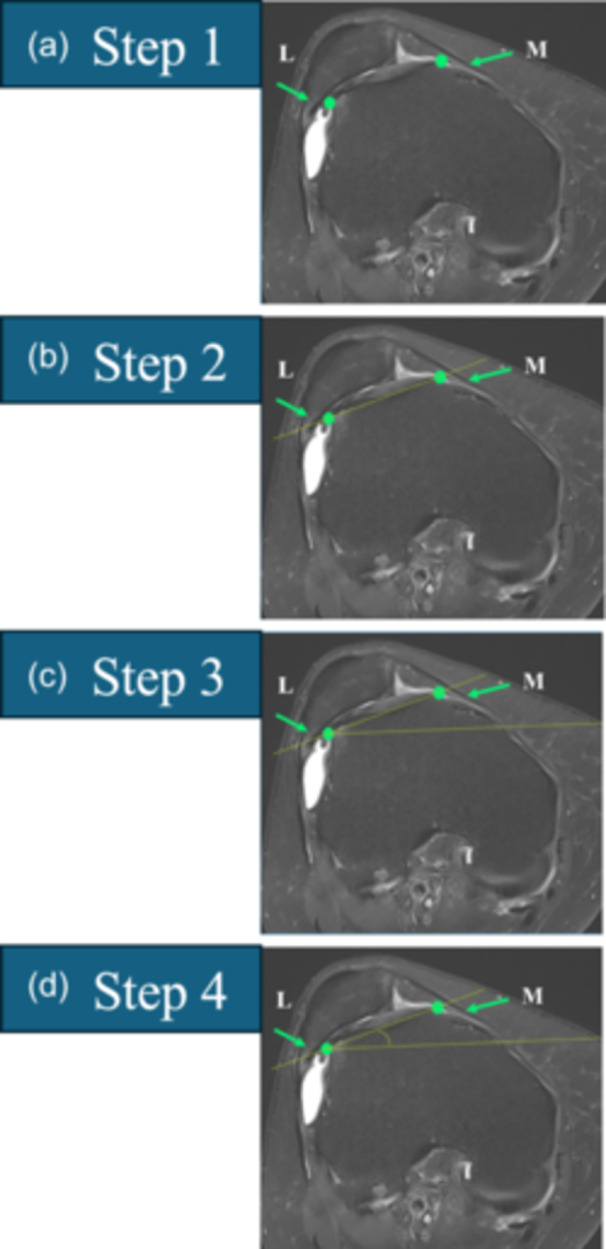
The cranial trochlear orientation (CTO) is measured in four steps. Start by scrolling from cranial to caudal and select the first slice in which the trochlear cartilage is clearly visible showing or covering at least the entire lateral facet. (a) Step 1: Digitize the most lateral (L) and most medial (M) points on the subchondral bone covered by cartilage (in case of doubts regarding these points, verify the presence of cartilage on the sagittal view). The subchondral bone is darker and more visible, unlike the rest of the cortex. This might help differentiate the cortex covered by cartilage from the cortex not covered. (b) Step 2: Draw a straight line (L–M) between L and M. (c) Step 3: Copy and paste the posterior bicondylar line (PBCL) and translate it anteriorly to intersect line L–M at point L. (d) Step 4: Measure the angle between lines L–M and PBCL. The angle is considered positive if point M is anterior to the PBCL. The angle is considered negative if point M is posterior to the PBCL.

**Figure 7:**
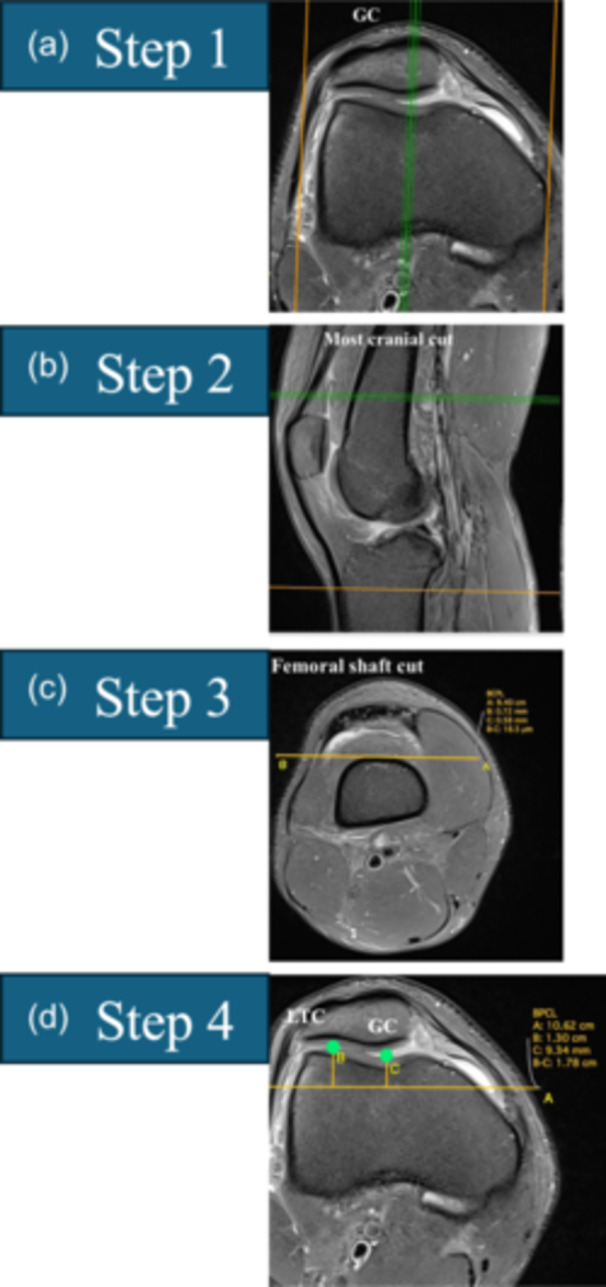
The axial plane central trochlear bump is selected in four steps. (a) Step 1: In the sagittal plane, select the slice that passes through the trochlear groove cartilage (TGC) and choose the most cranial axial slice. (b) Step 2: Copy and paste the posterior bicondylar line (PBCL) in the most cranial axial slice and translate it until it becomes tangent to the anterior margin of the femoral cortex. (c) Step 3: Copy and paste the translated PBCL in the axial slice that shows the TGC. (d) Step 4: The central prominence in the axial plane is the distance from the ‘translated PBCL’ to point TGC.

**Figure 8:**
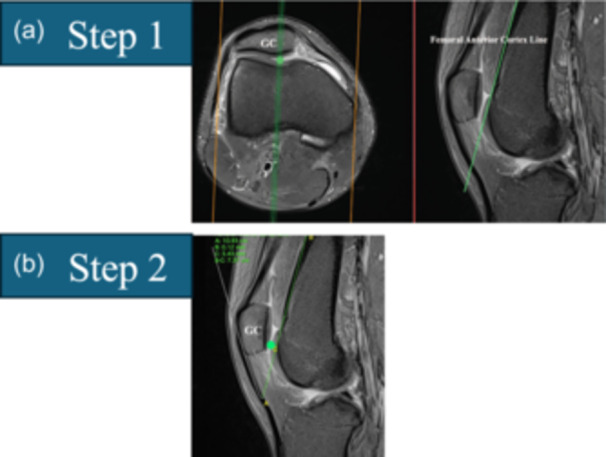
The sagittal plane central trochlear bump is selected in two steps. (a) Step 1: On the sagittal slice passing through the trochlear groove cartilage (TGC), draw the anterior femoral cortex line (AFCL) tangent to the anterior femoral cortex. (b) Step 2: The central prominence in the sagittal plane is the distance from the AFCL to the most anterior point on the cartilage, along the anteroposterior axis, passing through the TGC.

Four readers (*ED, TP, AG, CT; orthopaedic surgeons qualified, and trained in the reference centre*) independently made seven measurements (rounded to the nearest whole number) for all knees, using Horos™ (Horos Project):

(1) PBCL (Figure 2)
  The PBCL is defined in three steps. Step 1: Select the axial MRI slice that crosses the centre of the medial condyle. Step 2: Select the sagittal MRI slice that crosses the most posterior point of the medial condyle. Step 3: Draw the PBCL in the axial slice, which passes through the subchondral bone at the posterior aspect of the medial and lateral femoral condyles.
(2) Trochlear shape (Figure 3)
  Scrolling from cranial to caudal, the first slice is selected in which the trochlear cartilage is visible, showing/covering at least the entire lateral facet. The trochlear shape is described according to the cartilage contour as (a) concave (trochlea with a sulcus, as well as medial and lateral facets), (b) flat (trochlea with no evident sulcus nor medial facet) or (c) convex (trochlea with no sulcus nor medial facet, and domed lateral facet).
(3) Sulcus angle (Figure 4)
  Scrolling from cranial to caudal, the first slice is selected where the trochlear cartilage is visible, showing the sulcus formation and the medial facet. If the trochlea is concave or flat, the sulcus angle is measured in three steps (for convex trochleae, the sulcus angle is noted as ‘unmeasurable’). Step 1: the trochlear groove cartilage (TGC) point, the lateral trochlear cartilage (LTC) peak and the medial trochlear cartilage (MTC) peak are digitized. Step 2: A line (LTC–TGC) connecting points TGC and LTC and a line (TGC–MTC) connection points TGC and MTC are drawn. Step 3: The sulcus angle is measured as the angle between lines LTC–TGC and TGC–MTC.
(4) Lateral trochlear inclination (LTI) (Figure 5)
  Scrolling from cranial to caudal, the first slice is selected where the trochlear cartilage is visible, showing the sulcus formation and the medial facet. If the trochlea is concave or flat, the LTI is measured in four steps (for convex trochleae, the LTI is noted as ‘unmeasurable’). Step 1: The TGC point and the LTC peak are digitized. Step 2: A line (LTC–TGC) connecting points TGC and LTC is drawn. Step 3: Copy and paste the PBCL and translate it anteriorly to intersect line LTC–TGC at point TGC. Step 4: The LTI is measured as the angle between lines LTC–TGC and PBCL. The angle is considered positive if LTC is anterior to the PBCL. The angle is considered negative if LTC is posterior to PBCL.
(5) Cranial trochlear orientation (CTO) (Figure 6)
  Scrolling from cranial to caudal, the first slice is selected in which the trochlear cartilage is visible, showing/covering at least the entire lateral facet [12]. The CTO is measured in four steps. Step 1: The most lateral (L) and medial (M) points on the subchondral bone covered by cartilage are digitized. Step 2: A line (L–M) connecting point L and point M is drawn. Step 3: Copy and paste the PBCL and translate it anteriorly to intersect line L–M at point L. Step 4: The CTO is the angle between lines L–M and PBCL. The angle is considered positive if point M is anterior to the PBCL. The angle is considered negative if point M is posterior to the PBCL.
(6) Central trochlear bump in the axial plane (Figure 7)
  The axial plane central trochlear bump is selected in four steps. Step 1: In the sagittal plane, select the slice that passes through the TGC and choose the most cranial axial slice. Step 2: Copy and paste the PBCL in the most cranial axial slice and translate it until it becomes tangent to the anterior margin of the femoral cortex. Step 3: Copy and paste the translated PBCL in the axial slice that shows the TGC. Step 4: The central prominence in the axial plane is the distance from the ‘translated PBCL’ to point TGC.
(7) Central trochlear bump in the sagittal plane (Figure 8)

The sagittal plane central trochlear bump is selected in two steps. Step 1: On the sagittal slice passing through the TGC, draw the anterior femoral cortex line (AFCL) tangent to the anterior femoral cortex. Step 2: The central prominence in the sagittal plane is the distance from the AFCL to the most anterior point on the cartilage, along the anteroposterior axis, passing through the TGC.

### Agreement between readers

The four readers did an independent trial run on the same 20 knees to ensure they understood the measurement protocol. The agreement among readers to classify trochlear shape as concave, flat or convex was assessed using Gwet's agreement coefficient (Gwet's AC). The agreement for measuring sulcus angle, LTI, CTO and the central trochlear bump in the axial or sagittal plane was assessed using intraclass correlation coefficients (ICCs). Upon verification through discussion that all readers understood the protocol, each reader independently measured all knees. For the final data set of measurements, the agreement on classifying shape was substantial but not perfect classifying shape (Gwet's AC, 0.706), excellent for sulcus angle (ICC, 0.863), LTI (ICC, 0.853), central bump in the axial plane (ICC, 0.989) and the sagittal plane (ICC, 0.991) and good for CTO (ICC, 0.726) (Table 2). In the final analysis, the authors used the measurements of the most senior reader.

**Table 2:** Interobserver agreement of MRI measurements of shape, sulcus angle, LTI, CTO and central bump by four observers.

Variable	Test		Coefficient		95% C.I.		
Shape	Gwet's AC		0.706		0.648	–	0.764
Sulcus angle	ICC		0.863		0.828	–	0.892
LTI	ICC		0.853		0.815	–	0.886
CTO	ICC		0.726		0.644	–	0.791
Central bump							
Axial plane	ICC		0.989		0.978	–	0.995
Sagittal plane	ICC		0.991		0.981	–	0.996

Abbreviations: AC, agreement coefficient; C.I., confidence interval; CTO, cranial trochlear orientation; ICC, intraclass correlation coefficient; LTI, lateral trochlear inclination; MRI, magnetic resonance imaging.

### Statistical analysis

Continuous variables were summarized as means, standard deviation, ranges, medians and interquartile ranges. Categorical variables were summarized as frequencies and proportions. The statistical significance of differences in continuous variables between the OPI and control groups was evaluated using the Student's *t* test for normal distributions or Wilcoxon's rank‐sum test for nonnormal distributions. The effect size of differences was expressed as a mean difference and its 95% confidence interval (95% C.I.). The statistical significance of differences in categorical variables between the OPI and control groups was evaluated using the Kruskal–Wallis test. The effect size of differences was expressed as an odds ratio and its 95% C.I.

The three parameters that had excellent agreement (ICC > 0.800) were sulcus angle, LTI and central bump. The authors preferred to use central bump in the sagittal plane because of its similarity to the bump seen in true lateral x‐ray views of the original D. Dejour classification (v2.0). Thresholds for the three parameters were established using recursive partitioning and regression trees to distinguish between OPI and control knees. Recursive partitioning is a statistical method to split knees into groups with similar sulcus angle, LTI and central bump characteristics. Regression trees are a statistical method used to predict if a knee is part of the OPI or control group based on recursive partitioning. Finally, the authors evaluated the sensitivity and specificity of various combinations of sulcus angle and LTI to diagnose OPI. The combination that yielded the highest sensitivity was selected as a basis for classification, then the measurability of sulcus angle and LTI, as well as the presence of a central bump >5 mm were used to further grade the severity of trochlear dysplasia (Table 3).

**Table 3:** Sensitivity and specificity of various combinations of sulcus angle and LTI to diagnose OPI.

	Criteria to diagnose trochlear dysplasia	TP	FP	TN	FN	Sensitivity (%)	Specificity (%)
Option 1	Sulcus angle ≥157°	99	11	92	28	78	89
Option 2	LTI < 14°	108	26	77	19	85	75
Option 3	Sulcus angle ≥157° **AND** LTI < 14°	96	11	92	31	76	89
Option 4^a^	Sulcus angle ≥157° **OR** LTI < 14°	111	26	77	16	87	75

Abbreviations: FN, false negative; FP, false positive; LTI, lateral trochlear inclination; MRI, magnetic resonance imaging; OPI, objective patellar instability; TN, true negative; TP, true positive.

^a^ Option 4 retained for final MRI classification of trochlear dysplasia (Types 0–3).

A recent study by Gobbi et al. [14] compared 181 healthy knees with 142 knees having patellar instability and found that the sulcus angles were 148.3° ± 7.5° and 160.3° ± 11.4°, respectively, and the central trochlear bump heights were 3.6 ± 1.4 mm and 5.0 ± 1.8 mm, respectively. Based on these findings, to detect a standardized mean difference of 1.24 in the sulcus angle between healthy knees and knees with patellar instability, an a priori power sample size calculation using G*Power 3.1 [9] indicated that a total sample size of 36 knees (18 per group) would be needed to achieve a power of 95% with a 2.5% one‐sided significance level. Likewise, to detect a standardized mean difference of 0.923 in the central trochlear bump height between healthy knees and knees with patellar instability, an a priori power sample size calculation indicated that a total sample size of 64 knees (32/group) would be required to achieve a power of 95% with a 2.5% one‐sided significance level. All statistical analyses were performed using R Studio (2023.09.1 Build 494, Posit Software, PBC) and R (version 4.3.1, R Core Team [2023]. R: A Language and Environment for Statistical Computing. R Foundation for Statistical Computing.). The authors used the *ggplot2* [33], *ggpubr* [19], *reshape* [32], *irr* [10], *openxlsx* [28] and *rpart* [13] packages. A *p* < 0.05 was considered statistically significant.

## RESULTS

Differences in trochlear shape, sulcus angle, LTI, CTO and the central bump were statistically significant (*p* < 0.001) between the OPI and the control groups. The sulcus angle was larger in the OPI group (160° ± 7.6°) compared to the control group (149.2° ± 6.1°) with a mean difference of 10.8° (95% C.I. 8.8°–12.8°). The LTI was smaller in the OPI group (11.3° ± 4.3°) compared to the control group (16.7° ± 3.7°) with a mean difference of 5.4° (95% C.I. 8.8°–12.8°). The sagittal plane central bump was higher in the OPI group (6.0 ± 2.2 mm) compared to the control group (2.8 ± 1.8 mm) with a mean difference of 3.2 mm (95% C.I., 2.7–3.7 mm) (Table 4).

**Table 4:** Comparative statistics of trochlear shape, sulcus angle, LTI, CTO and central bump between knees with OPI and control knees.

	OPI (*n* = 127)		Control (*n* = 103)	
	Mean ± SD; *n* (%)	Range	*Median*	*IQR*	Mean ± SD; *n* (%)	Range	*Median*	*IQR*	*p* Value	Effect size		95% C.I.
Trochlear shape												
Concave	16 (13)				81 (79)				<0.001	OR	0.04	0.02–0.08
Flat	68 (54)				20 (19)					OR	4.8	2.6–8.7
Convex	43 (34)				2 (2)					OR	25.9	6.1–110
Sulcus angle, deg	160.0 ± 7.6	140–180	*160*	*155*–*165*	149.2 ± 6.1	134–167	*149*	*145*–*153*	<0.001	MD	10.8	8.8–12.8
LTI, deg	11.3 ± 4.3	0–20	*11*	*9*–*14*	16.7 ± 3.7	9–29	*17*	*14*–*19*	<0.001	MD	−5.4	−6.6–4.2
CTO, deg	−2.3 ± 8.3	−38–22	*−3*	*−7–2*	−10.8 ± 5.1	−26–−1	*−11*	*−14–−7*	<0.001	MD	8.6	6.8–10.3
Central bump												
Axial plane, mm	6.0 ± 2.1	1–12	*6*	*5*–*8*	2.8 ± 1.9	−2–8	*3*	*1*–*4*	<0.001	MD	3.2	2.7–3.7
Sagittal plane, mm	6.0 ± 2.2	1–12	*6*	*5*–*8*	2.8 ± 1.8	−2–8	*3*	*1*–*4*	<0.001	MD	3.2	2.7–3.7

Abbreviations: C.I., confidence interval; CTO, Cranial trochlear orientation; deg, degrees; IQR, interquartile range; LTI, lateral trochlear inclination; MD, mean difference; mm, millimetre; OPI, objective patellar instability; OR, odds ratio; SD, standard deviation.

**Figure 9:**
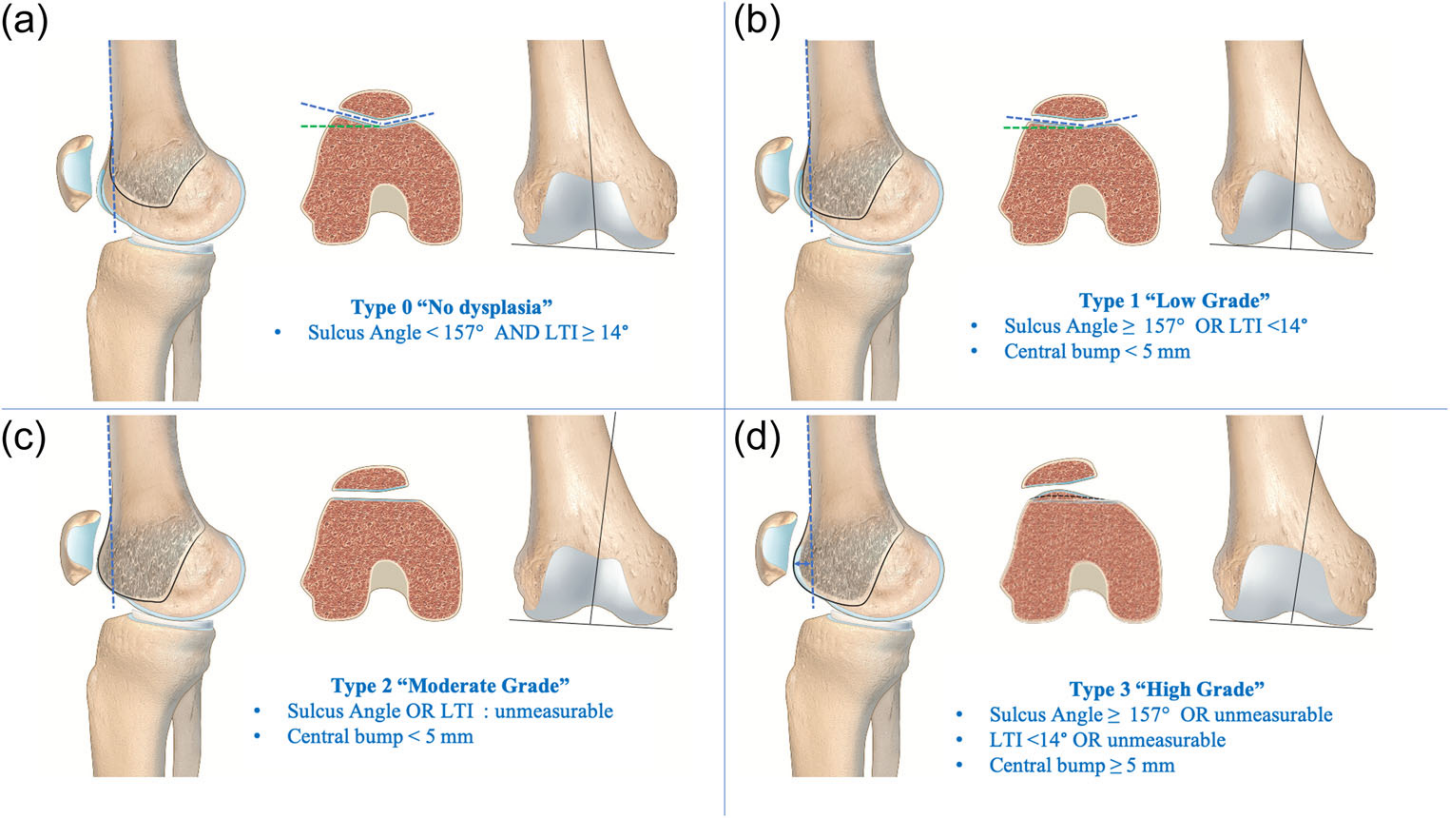
The quantitative magnetic resonance imaging classification (v3.0) can be summarized as: (a) Type 0 (No trochlear dysplasia): sulcus angle <157° **AND** lateral trochlear inclination (LTI) ≥ 14°. (b) Type 1 (Low‐grade trochlear dysplasia): (sulcus angle ≥157° **OR** LTI < 14°) **AND** central bump <5 mm. (c) Type 2 (Moderate‐grade trochlear dysplasia): (sulcus angle ‘unmeasurable’ **OR** LTI ‘unmeasurable’) **AND** central bump <5 mm. (d) Type 3 (High‐grade trochlear dysplasia): (sulcus angle ≥157° **OR** ‘unmeasurable’ **OR** LTI < 14° **OR** ‘unmeasurable’) **AND** central bump ≥5 mm.

The recursive partitioning and regression tree models established thresholds of ≥157° for sulcus angle, <14° for LTI and ≥5 mm for the central bump. The highest sensitivity (87%) for diagnosing OPI was achieved with the combination of ‘Sulcus angle ≥157° **OR** LTI < 14°’ (Table 3). This combination was then utilized to further grade trochlear dysplasia based on whether sulcus angle **OR** LTI were noted as ‘unmeasurable’, and on the presence of a central bump ≥5 mm. The selected quantitative MRI classification is summarized below (Figure 9):

**Type 0 (No trochlear dysplasia)**

- sulcus angle <157° **AND** LTI ≥ 14°

**Type 1 (Low‐grade trochlear dysplasia)**

- (sulcus angle ≥157° **OR** LTI < 14°) **AND** central bump <5 mm

**Type 2 (Moderate‐grade trochlear dysplasia)**

- (sulcus angle **OR** LTI ‘unmeasurable’) **AND** central bump <5 mm

**Type 3 (High‐grade trochlear dysplasia)**

- (sulcus angle ≥157° **OR** ‘unmeasurable’ **OR** LTI < 14° **OR** ‘unmeasurable’) **AND** central bump ≥5 mm

**Figure 10:**
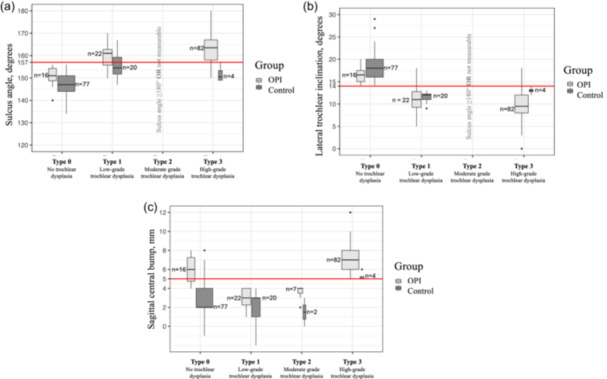
Comparison of the sulcus angle (a), lateral trochlear inclination (b) and central bump (c) among knees with a Type 0, 1, 2 or 3 trochlear dysplasia classification.

The classification identified 93 knees as ‘Type 0’, 42 as ‘Type 1’, 9 as ‘Type 2’ and 86 as ‘Type 3’ (Table 5). As expected, there was a linear progression in the prevalence of OPI from Type 0 (16 knees, 17.2%) to Type 1 (22 knees, 52.4%), Type 2 (7 knees 77.8%) and Type 3 (82 knees, 95.3%) (Figure 10, Table 5).

**Table 5:** Comparison of sulcus angle, LTI and central bump between knees with OPI and control knees classified as Type 0, 1, 2 or 3.

	OPI		Control	
	Mean ± SD	Range	Mean ± SD	Range
Type 0—no trochlear dysplasia (*n* = 93)	16 (17.2%)		77 (82.8%)	
Sulcus angle, deg	150.9 ± 4.3	140–156	147.5 ± 5.1	134–156
LTI, deg	16.8 ± 2.0	14–20	18.2 ± 2.9	14–29
Central bump in sagittal plane, mm	5.9 ± 1.5	4–8	2.8 ± 1.9	−1–8
Type 1—low‐grade trochlear dysplasia (*n* = 42)	22 (52.4%)		20 (47.6%)	
Sulcus angle, deg	159.7 ± 5.4	150–170	155.6 ± 5.6	147–167
LTI, deg	11.0 ± 3.0	5–18	11.7 ± 1.1	9–13
Central bump in sagittal plane, mm	3.0 ± 0.9	1–4	2.3 ± 2.3	−2–4
Type 2—moderate‐grade trochlear dysplasia (*n* = 9)	7 (77.8%)		2 (22.2%)	
Sulcus angle, deg	Unmeasurable		Unmeasurable	
LTI, deg	Unmeasurable		Unmeasurable	
Central bump in sagittal plane, mm	3.6 ± 0.8	2–4	1.5 ± 2.1	0–3
Type 3—high‐grade trochlear dysplasia (*n* = 86)	82 (95.3%)		4 (4.7%)	
Sulcus angle, deg^a^	163.3 ± 6.9	150–180	151.8 ± 3.8	149–157
LTI, deg^a^	9.5 ± 3.8	0–18	13.0 ± 0.8	12–14
Central bump in sagittal plane, mm	7.1 ± 1.6	5–12	5.3 ± 0.5	5–6

Abbreviations: deg, degrees; LTI, lateral trochlear inclination; mm, millimetre; OPI, objective patellar instability; SD, standard deviation.

^a^ Sulcus angle and LTI unmeasurable in 36 knees.

## DISCUSSION

The present study adapted the current D. Dejour trochlear dysplasia classification (v2.0) to use MRI as the only imaging modality (v3.0). This MRI classification depended exclusively on quantitative measurements, which had an excellent interobserver agreement and yielded very high sensitivity to diagnose OPI. The MRI imaging protocol combined with the MPR mode and standardized measurement methodology are mandatory to ensure objective and repeatable grading of trochlear dysplasia and to determine the ideal treatment plan. The clinical relevance is that this MRI classification for trochlear dysplasia, based purely on quantitative parameters, could be quickly adopted and correctly applied by clinicians worldwide in any type of institution.

Trochlear dysplasia is the primary risk factor for patellar instability [4, 30], and it is therefore crucial to use a reliable and accurate classification, to select ideal treatment plans and clinical decision‐making. Recently, two quantitative parameters have been published defining the orientation of the whole trochlea with the CTO [12] and the abnormal medial orientation of the groove in high‐grade trochlear dysplasia [22] using quantitative parameters on MRI. The presented quantitative MRI‐based classification system can be used to implement a menu à la carte treatment for patients with OPI, in addition to mandatory medial patellofemoral ligament reconstruction. In Types 0 and 1, surgery can be performed to correct excessive patellar height or tibial tubercle–trochlear groove distance (TT‐TG) if necessary, without any procedures on the trochlea. In Type 2, surgery can be performed to correct excessive patellar height or TT‐TG if required, but a distalizing tibial tubercle osteotomy can be considered, even in cases with normal patellar height, to improve patellofemoral congruence by engaging the patella in a deeper region of the trochlear groove. In Type 3, a sulcus‐deepening trochleoplasty should be considered to remove the bump, increase patellofemoral congruence and correct excessive patellar height or TT‐TG if necessary. Therefore, an adapted therapeutic proposal can be suggested for each dysplasia type of the new classification system. This is in contrast to the D Dejour system (v2.0) for which specific surgical treatments could be proposed for Types B and D (trochleoplasty).

The D. Dejour classification remains the most widely used system to assess trochlear dysplasia. Still, studies use a variety of imaging protocols in different combinations and apply nonstandardized criteria to distinguish severe from moderate dysplasia due to the difficulty of acquiring true lateral radiographs [26]. Although most report moderate to near‐perfect interobserver agreement for the D. Dejour classification [26], a recent study by Martinez‐Cano et al. [21] revealed only slight interobserver agreement between 28 patellofemoral experts who graded trochlear dysplasia using true lateral radiographs and MRI. Jian‐Lüssi et al. [18] proposed an adapted scheme of the D. Dejour classification using only axial and sagittal MRI and reported substantial interobserver agreement for two readers that classified Types A to D. Falkowski et al. [8] proposed an adapted version of the Hepp classification using axial MRI and reported moderate interobserver agreement for two readers who classified Types 1–5. Sharma et al. [29] proposed the Oswestry‐Bristol classification using axial MRI and reported fair to good interobserver agreement among four readers who classified trochleae qualitatively as normal, shallow, flat or convex and did not consider the presence and size of bump. Training and discussion on how to apply a classification in small groups could increase agreement; however, it is unfeasible for larger groups and across settings, emphasizing the need for unambiguous classification tools. The present study proposes an unambiguous classification tool since it is based on quantitative measurements using a single imaging modality for which optimal orientation is ensured independent of patient position during the scan.

The aforementioned qualitative classification systems are based on assessment of morphologic features that rely on the reader's subjective interpretation to describe trochlear shape. Martinez‐Cano et al. [21] suggested that poor agreement between observers may be due to key factors being assessed qualitatively as present or absent; for example, if the trochlear bump is not measured, one surgeon may consider it small or insignificant, whereas another may consider it big or significant, regardless of its actual size. Nacey et al. [24] reported that the D. Dejour classification system accurately identifies trochlear dysplasia and can differentiate between low‐grade (Type A) and high‐grade dysplasia (Type B, C and D), but that quantitative measures are needed to differentiate between Types B and C and between Types B and D. The present study revealed moderate to substantial agreement among four readers that classified trochlear shape (concave, flat or convex) but excellent agreement for morphological measurements (sulcus angle, LTI and the central bump). These morphological measurements have been incorporated into a decision tree to classify trochlear dysplasia as none (Type 0), low‐grade (Type 1), moderate‐grade (Type 2) or high‐grade (Type 3), which solely relies on objectively comparing measurements with thresholds. Moreover, only one imaging modality—MRI—is used for which the imaging protocol is clearly defined, and the measurement protocol is standardized by clearly describing the method to make the measurements and defining the slices on which measurements are taken. This classification system (v3.0) allows quantitative assessment of trochlear morphology in a linear manner from low‐grade, moderate‐grade, to high‐grade rather than the previous classification (v2.0) where Types B and D indicated higher grades of dysplasia than Types A and C.

The classification system in the present study relies on measuring three trochlear features–sulcus angle, LTI and central bump–on MRI. To ensure reliable quantification of the bump height, the central bump should be measured on a true lateral view, which can be achieved using the MPR mode. It supposes that the prescription of an MRI for patellofemoral disorders includes the native acquisition with a sufficient distance above the trochlea and below the tibial tubercule. These measurements could be tedious and time‐consuming, especially for surgeons and radiologists with less experience. Moreover, incorrect application of the measurement protocol could lead to misdiagnosis. Artificial intelligence has been successfully used to detect meniscal tears [15, 20, 36], anterior cruciate ligament injury [2, 17, 23] and the diagnosis of knee osteoarthritis [7, 25, 35]. Recently Xu et al. [34] proposed an aided diagnosis algorithm framework based on deep‐learning technology that distinguished normal trochleae from dysplastic trochleae with the same reliability as that of senior surgeons (ICC = 0.91, 95% CI: 0.87–0.94) but at an average time of only 0.14 s per knee. Since the present classification system only relies on quantitative assessment, it would be ideal for implementation in a deep‐learning model.

The present study's findings should be interpreted with the following considerations and limitations. First, the thresholds for sulcus angle, LTI and bump are derived from a cohort of knees representative of mainly one European country that underwent MRI at one centre. In addition, the established thresholds and classification system must be validated on independent data sets from other centres and countries. Second, interobserver agreement was excellent for sulcus angle, LTI and bump, but this was between four experienced readers. Third, the classification system only considers the most important risk factor for OPI, that is, trochlear dysplasia. As a result, 16 knees with OPI were classified as Type 0. These knees all had a sagittal patellofemoral engagement (SPFE) < 35% (functional patellar alta). The MRI patellar height index was over 1.1 (1.2 for five of the 16), and the sulcus angle was between 149° and 156° (borderline Type 1). Therefore, these patients have a risk factor for patellar dislocation due to the high‐riding patella with a low SPFE. Fourth, the sensitivity and specificity of the quantitative MRI‐based classification system were not compared to that of other qualitative classification systems. Finally, thresholds were not based on a sex‐specific analysis to derive separate thresholds for men and women. A recent study demonstrated that normal values and thresholds for patellar instability could vary between men and women [30].

## CONCLUSION

This MRI classification depends exclusively on quantitative measurements, has excellent interobserver agreement, and yields very high sensitivity to diagnose OPI. The MRI imaging protocol combined with the MPR mode and standardized measurement methodology could be quickly adopted and correctly applied by clinicians worldwide in any type of institution to determine the ideal treatment plan.

## AUTHOR CONTRIBUTIONS

**David H. Dejour**: Conceptualization; investigation; methodology; validation; writing—review and editing. **Edoardo Giovanetti de Sanctis**: Data curation; investigation; methodology. **Jacobus H. Müller**: Methodology; software; writing—original draft. **Etienne Deroche**: Investigation; methodology. **Tomas Pineda**: Investigation; methodology. **Amedeo Guarino**: Investigation; methodology. **Cécile Toanen**: Investigation; methodology. **Patellofemoral Imaging Group**: Investigation; methodology; writing—original draft.

## CONFLICT OF INTEREST STATEMENT

D. D. reports personal fees from SBM, outside the submitted work. The remaining authors declare no conflict of interest.

## ETHICS STATEMENT

The institutional review board approved the study in advance (IRB reference number: COS‐RGDS‐2023‐11‐008‐DEJOUR‐D). All patients provided written informed consent to use their data and images for research and publishing purposes.

## Data Availability

Upon reasonable request, the authors can provide access to the data used for all analyses and analytic code.
